# Association between Micronutrients and Hyperhomocysteinemia: A Case-Control Study in Northeast China

**DOI:** 10.3390/nu15081895

**Published:** 2023-04-14

**Authors:** Can Sun, Ding Ding, Zhouyu Wen, Chengmei Zhang, Juan Kong

**Affiliations:** Department of Clinical Nutrition, Shengjing Hospital of China Medical University, Shenyang 110004, China

**Keywords:** hyperhomocysteinemia, homocysteine, *MTHFR* C677T polymorphism, zinc

## Abstract

Hyperhomocysteinemia (HHcy) is an independent risk factor for cardiovascular and cerebrovascular diseases where the plasma homocysteine (Hcy) concentration exceeds 15 µmol/L. HHcy is affected by vitamins B12, B6, and folic acid (fol); however, its relationship with other nutrients is not fully understood. We investigated the nutritional and genetic factors associated with HHcy and the possible dose–response relationships or threshold effects in patients in Northeast China. Genetic polymorphisms and micronutrients were tested with polymerase chain reaction and mass spectrometry, respectively. This trial was registered under trial number ChiCTR1900025136. The HHcy group had significantly more males and higher body mass index (BMI), methylenetetrahydrofolate reductase (*MTHFR* 677TT) polymorphism proportion, and uric acid, Zn, Fe, P, and vitamin A levels than the control group. After adjusting for age, sex, BMI, vitamin B12, fol, and *MTHFR* C677T, the lowest Zn quartile reduced the odds ratio of HHcy compared with the highest Zn quartile. The dose–response curves for the association between plasma Zn and HHcy were S-shaped. High plasma Zn concentrations were significantly correlated with high HHcy odds ratios, and the curve leveled off or slightly decreased. Most importantly, HHcy risk decreased with decreasing plasma Zn concentration; the threshold was 83.89 µmol/L. Conclusively, individuals residing in Northeast China, especially those with the *MTHFR* 677TT polymorphism, must pay attention to their plasma Zn and Hcy levels.

## 1. Introduction

Homocysteine (Hcy) is a sulfur-containing amino acid and an intermediate metabolite of methionine and cysteine, mainly through remethylation and sulfuration [1]. In the physiological state, Hcy levels in the body are maintained at 5–15 µmol/L [2]. Many factors, such as heredity, medicine use, diseases, and living habits, may lead to increased Hcy levels (>15 µmol/L), resulting in hyperhomocysteinemia (HHcy) [3]. HHcy is considered a cytotoxic factor associated with various diseases such as coronary heart disease, stroke, Alzheimer’s disease, peripheral vascular disease, cancer, diabetes, and osteoporosis [4,5,6]. Furthermore, numerous epidemiological reports have established HHcy as an independent risk factor for cardiovascular disease, cerebrovascular disease, dementia-type disorders, and associated fractures [7,8,9].

During remethylation, Hcy can be methylated twice via two different pathways. First, methylenetetrahydrofolate reductase (*MTHFR*) reduces 5,10-methyltetrahydrofolate (*MTHF*) to form 5-*MTHF* [10]. Then 5-*MTHF*, assisted by the cofactor vitamin B12, adds a methyl group to Hcy, which again produces methionine. This requires the key enzyme methionine synthase reductase (*MTRR*) [11,12].

More than 40 polymorphism sites are present in *MTHFR* [13], of which C677T and A1298C are the most impactful [14]. Compared with the 677CC genotype, the 677TT genotype shows approximately 34% *MTHFR* activity, while the heterozygote 677CT shows approximately 65% *MTHFR* activity [15]. In *MTRR*, the most important polymorphism is A66G [16], which regulates Hcy levels through DNA hypomethylation [17]. Polymorphisms differ between distinct geographical areas and ethnic populations [18,19,20].

Folic acid (fol) is a water-soluble vitamin involved in nucleic acid synthesis, DNA methylation, repair, cell division, and embryonic development [21,22,23]. Hcy remethylation and trans-sulfur pathways require vitamins B12 and B6 as coenzymes, respectively. A two-month-long, randomized, double-blind study found that vitamin D supplementation decreased hypertension and reduced Hcy levels [24].

Minerals also greatly influence the occurrence of diseases; for example, Zn [25], Fe [26], and Mg [27] are related to atherosclerosis and vasospasm [28]. Further, minerals are involved in the complex physiological reactions in the body, serving as components of some metabolic enzymes or as auxiliary factors regulating enzyme activity. For example, Zn regulates the activity of more than 100 enzymes [28,29]. However, there are few studies that have elucidated whether minerals are relevant factors influencing Hcy metabolic enzymes. Sadako Matsui’s [30] cross-sectional study showed that Zn was negatively correlated to log Hcy in men, but not in women. Esfandiar Heidarian [31] conducted a randomized, double-blind, controlled, crossover study which showed that Zn supplementation reduced serum Hcy and increased vitamin B12 and fol concentrations in T2DM patients with microalbuminuria. However, Véronique Ducros [32] showed that Zn supplementation did not modify Hcy, vitamin B12, or RBC fol values in aging healthy people.

Therefore, in this study, we aimed to investigate the relationship between plasma Hcy levels and genetic variation in *MTHFR* and *MTRR* in patients from Northeast China. In addition, as several vitamins play key roles in the metabolism of Hcy, we studied the relationship between vitamin levels and HHcy. Moreover, we explored the nutritional and genetic factors associated with HHcy and the potential dose–response relationships and threshold effects.

## 2. Materials and Methods

### 2.1. Study Participants

From June 2019 to December 2020, according to the inclusion and exclusion criteria, 203 participants were enrolled at the clinical nutrition department of Shengjing Hospital, China Medical University (Shenyang, China). Specific study inclusion requirements were no serious organic disease, independent mobility, clear awareness, and voluntary participation. Exclusion criteria were severe organ lesions, immobility, and unconsciousness. No age or sex restrictions were laid. All participants were tested for HHcy through the enzyme cycle method and divided into the normal (plasma Hcy 5–15 µmol/L) and HHcy (plasma Hcy > 15 µmol/L) groups [14]. Then, 135 healthy individuals (control group) and 68 patients (HHcy group) were included in the study. This study fulfilled the principles of the Declaration of Helsinki and was approved by the ethics committee at Shengjing Hospital of China Medical University, China, and registered with a trial number ChiCTR1900025136. All participants received written informed consent to use their clinical data for research purposes.

### 2.2. Experimental Instruments and Reagents

Fasting venous samples (5 mL) were collected from all subjects in the morning, and plasma was centrifuged after anticoagulant treatment (3000 rpm for 15 min). A nucleic acid extraction reagent (EE201-01) was purchased from TransGen Biotech (Beijing, China) and a human *MTHFR* gene detection kit was purchased from Wuhan Youzhiyou Medical Technology (Wuhan, China). Polymerase chain reaction (PCR) amplification devices (7500-fast, ABI, Alameda, CA, USA) were used in this study. Hcy and other nutrients were completed in the clinical laboratory of Shengjing Hospital according to clinical testing standards. Plasma Hcy levels were determined through chemiluminescence using a commercial kit (Jiuqiang Biological Company, Beijing, China) and an automatic chemiluminassay analyzer (Abbott-i16200, Shanghai, China), following the detection methods reported by Yin [33,34]. Plasma Zn, Cu, Ca, P, Mg, and Pb levels were determined using an atomic absorption spectrometer (Jingbohui Biotechnology Company, Beijing, China), following the method by Komarova [35,36]. Plasma vitamin A, vitamin D, fol, and vitamin B12 levels were determined using an automatic electrochemiluminescence immune analyzer (Roche, Cobas e601E-E, Shanghai, China), following the method by Stokes [37,38,39] and its companion Detection special kit, in strict accordance with the Electrochemistry Luminescence method, instrument operating specifications, and detection kit instructions.

### 2.3. Sample Collection, DNA Preparation, and MTHFR Genotyping

**Figure 1:**
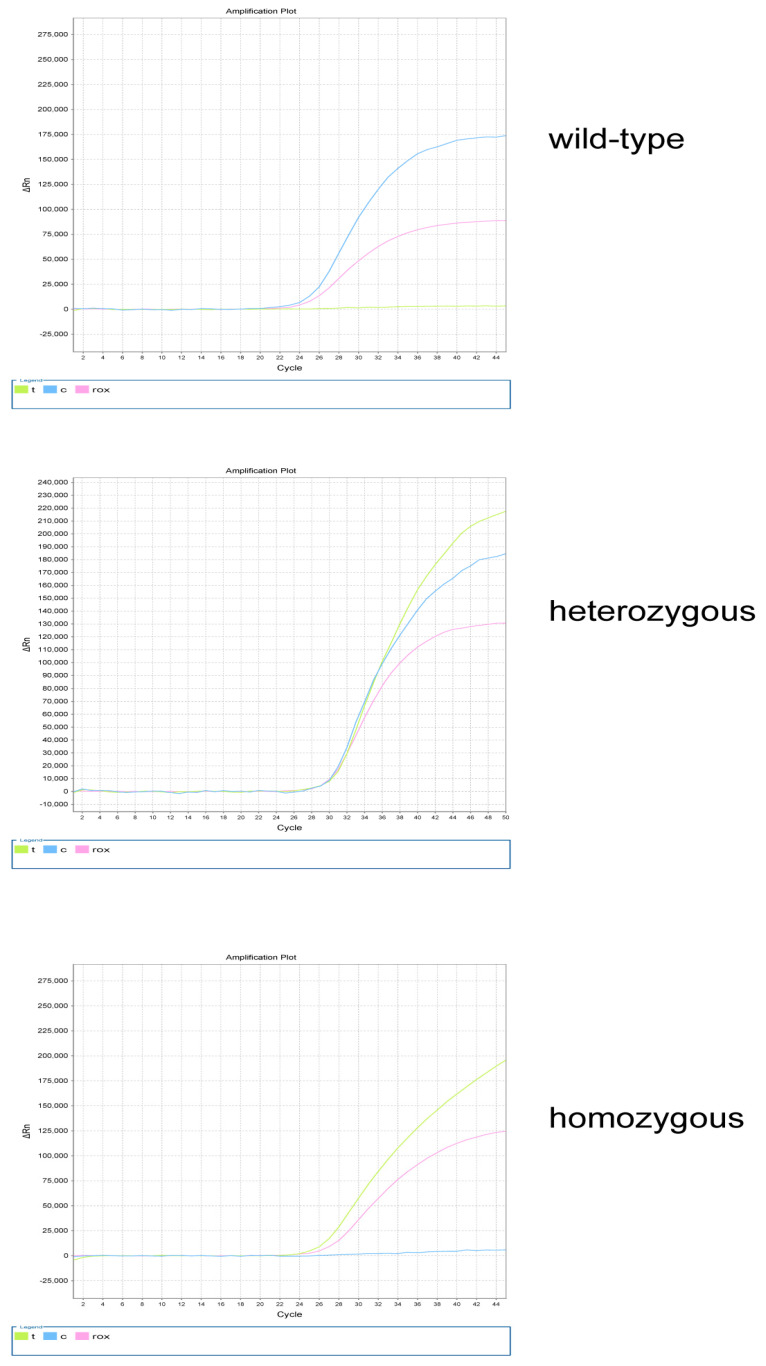
*MTHFR* genotyping. The wild−type genotypes include *MTHFR* 677CC, *MTHFR* 1298AA, and *MTRR* 66AA; the heterozygous genotypes include *MTHFR* 677CT, *MTHFR* 1298AC, and *MTRR* 66AG; and the homozygous genotypes include *MTHFR* 677TT, *MTHFR* 1298CC, and *MTRR* 66GG; t represents the FAM fluorescence channel, c represents the VIC fluorescence channel, and ROX represents internal reference.

The exfoliated cells were collected with disposable oral swabs and subjected to DNA extraction using a commercial DNA extraction kit, following the manufacturers’ instructions. The extracted DNA was stored at −20 °C. The PCR amplification conditions were 37 °C for 10 min, 95 °C for 5 min, 95 °C for 15 s (40 cycles), and 60 °C for 60 s. The fluorescence channel settings used were FAM, VIC, and ROX. According to the comparison of the two fluorescence signal intensities, the genotype was determined as wild-type, heterozygous, or homozygous (Figure 1).

### 2.4. Statistical Analysis

The statistical software SPSS 20 (IBM Corp., Armonk, NY, USA) was used for data processing and statistical analyses. Results with *p* < 0.05 were considered significant. The chi-square or Fisher’s exact tests were used for categorical variables, and a one-way analysis of variance was used for continuous variables. Odds ratios (ORs) with 95% confidence intervals were estimated using logistic regression analysis. We also used restricted cubic splines (RCSs) to test for linearity and explore the shape of the dose–response effect between plasma Zn concentration and HHcy.

## 3. Results

### 3.1. Participant Characteristics

The characteristics of the 203 participants are listed in Table 1. According to the criteria of plasma Hcy concentration >15 µmol/L, 68 patients had HHcy (HHcy group). Additionally, 135 healthy individuals (control group) were included. The average plasma Hcy concentration was 35.89 and 9.26 µmol/L in the HHcy and control groups, respectively. The HHcy group had more male and overweight/obese participants and showed higher alkaline phosphatase (ALKP) and uric acid (UA) levels than the control group. The *MTHFR* C677T mutation distribution was significantly different between the groups (*p* < 0.001). The HHcy group had more proportion of participants with *MTHFR* 677TT type than the control group (51.50 vs. 22.20%). There was no significant difference in *MTHFR* 1298 and *MTRR* 66 mutations between the groups (*p* = 0.083 and *p* = 0.853, respectively).

### 3.2. Comparison of Hcy Concentration among Different Genotypes

Table 2 shows an overall comparison of Hcy concentrations for different genotypes and a comparison of Hcy concentrations in gender subgroups. The results of all Hardy–Weinberg tests were *p* > 0.05. Hcy levels were significantly higher in the *MTHFR* 677TT group than in the *MTHFR* 677CC or *MTHFR* 677CT groups. This difference was significant in the total and male groups (*p* < 0.001), but not statistically significant in the female group (*p* = 0.095). However, there were no significant differences in the Hcy levels in the wild-type, heterozygous, and homozygous *MTHFR* A1298C and *MTRR* A66G groups, indicating that Hcy levels were affected by the *MTHFR* C677T polymorphism, instead of *MTHFR* A1298C or *MTRR* A66G.

### 3.3. Nutrient Differences among Participants

Table 3 shows the nutrient differences between the control and HHcy groups. The HHcy group had significantly higher plasma Zn, Fe, and vitamin A levels than the control group. In addition, the HHcy group had significantly lower plasma fol, vitamin B12, and P levels than the control group. There were no significant differences in plasma vitamin D, Pb, Cu, Ca, and Mg levels between the two groups.

### 3.4. Associations between Zn and HHcy ORs

Table 4 shows the associations between Zn and HHcy ORs. Plasma Zn levels were divided into four quartiles. Model 1 was an unadjusted model; Model 2 was adjusted for age, sex, and body mass index (BMI); and Model 3 was further adjusted for vitamin B12, fol, and *MTHFR* C677T. All three models indicated that Q1 had a significantly lower HHcy risk than Q4.

### 3.5. Dose–Response Association between Plasma Zn Level and HHcy Risk

**Figure 2:**
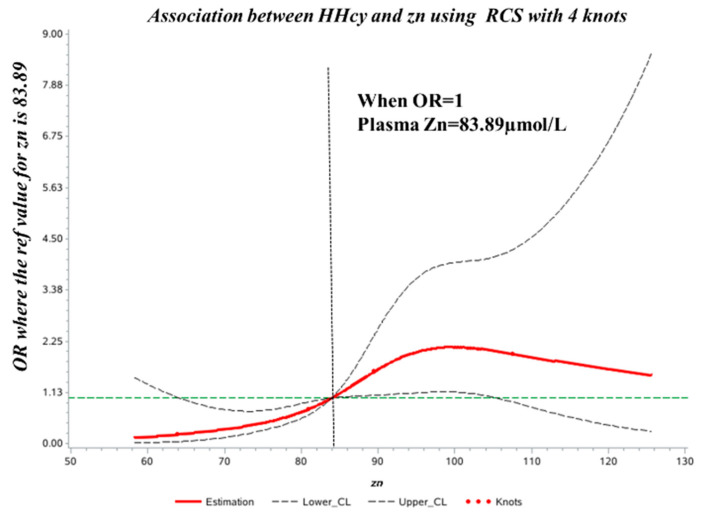
Dose–response relationship between plasma Zn concentration and HHcy odds ratio (OR). The ORs (solid lines) and 95% confidence intervals (dashed lines) of plasma Zn concentration are indicated by straight lines based on restrictive cubic splines. Age, sex, vitamin B12, folic acid, and *MTHFR* C677T were adjusted.

The spline regression model showed that the HHcy OR increased significantly when plasma Zn concentration was >83.89 µmol/L. Correspondingly, when plasma Zn was <83.89 µmol/L, the HHcy OR decreased significantly (Figure 2).

## 4. Discussion

The incidence of hypertension and cardiovascular diseases in Shenyang is higher than that in other cities in China [40]. HHcy is an independent risk factor for cardiovascular and cerebrovascular diseases, but studies on its relationship with nutrients are scarce. Therefore, using clinical data from a large tertiary care hospital, we investigated the genetic and nutritional factors associated with HHcy.

We observed that more males were in the HHcy group than in the control group, as in the study by Yating Yang [41]. They enrolled 330 Han Chinese patients with schizophrenia (SZ) and 190 healthy controls and found that male sex and older age were independent risk factors for HHcy in patients with SZ. This sex-related difference may have been caused by genetic factors or hormone levels. Similarly, our study showed the HHcy group to be older than the control group, but not significantly.

In addition to sex and age, the HHcy group showed a higher BMI. Kittisak Thawnashom [42] included 149 Thai overweight/obese and 113 control participants to analyze the association between the *MTHFR* C677T polymorphism and plasma Hcy, fol, and vitamin B12 concentrations. They found that the overweight/obese group had higher Hcy levels than the control group. Furthermore, Gallistl [43] enrolled 84 children and adolescents to assess the association among plasma Mg concentration, the *MTHFR* C677T mutation, and metabolic risk factors for coronary heart disease (CHD). Their results also showed that after adjusting for age and sex, Hcy levels were significantly correlated with BMI.

The prevalence of *MTHFR* C677T mutation was significantly higher and that of *MTHFR* A1298C was lower in our study than in Boyi Yang’s [44] study. Additionally, there was little difference in the prevalence of *MTRR* A66G between the two studies. It should be noted that the Hcy levels in *MTHFR* 1298CC homozygous mutants were significantly lower than that in the wild-type 1298AA. Similar to the results from Oliveira’s study [45], the Hcy concentration in the *MTHFR* 1298 CC group decreased by 10% in males and by 5% in females, compared to that in the *1298AA* group. In addition, we obtained the same result as Zappacosta [46]: the Hcy value was higher in the wild-type (1298 AA) group than in the homozygote mutation (1298 CC) group. In our study, although the *MTHFR* TT polymorphism significantly contributed to an increase in Hcy concentration, there were still many individuals with *MTHFR* TT in the group with normal Hcy, which should be reflected in the real value of gene detection and the interaction between gene and environment. Individuals with gene mutations should pay special attention to their daily diet and even take supplements to obtain healthy levels of vitamin B, especially at a young age. This way, they could avoid having high blood Hcy levels for several years without knowing, which can eventually lead to the occurrence of disease. Therefore, to reduce the incidence of HHcy-related diseases, nutritional and lifestyle improvements should be implemented in young children with genetic mutations.

In addition to the gene mutations mentioned above, micronutrients and vitamins play a basic role in regulating the metabolism of Hcy as enzyme cofactors. Our results showed that Zn, which is essential for the activity of many enzymes as an intracellular ion, was present in higher levels in the HHcy group than in the control group. Methionine synthase (MTR) and BHMT are Zn-dependent methyltransferases that participate in the remethylation of Hcy [47]. The high Zn content in the HHcy group in this study may be because the diet of the patients was mainly composed of meat containing high levels of purine and Zn. In their mother–infant pair-based study, Dilli Det [48] enrolled 108 newborns with CHD and 103 healthy newborns. They found that high levels of Hcy and Zn with low levels of vitamin D might be involved in the pathogenesis of CHD. Hector Vázquez-Lorente [49] recruited 51 healthy postmenopausal woman volunteers to take a 50 mg/day Zn supplement or placebo for eight weeks to assess the effect of Zn on plasma Hcy concentration. They confirmed that Zn supplementation enhanced plasma fol and Hcy levels.

There are relatively few studies on Zn and HHcy and examining the dose–response relationship or threshold effect between them. Regardless, the problem of excess Zn has been studied extensively in recent years. Abigail-Podany [50] used neonatal mice to test the effects of excess dietary Zn on intestinal function and host—microbe interactions during early life. The authors found that excess Zn in the diet causes oxidative stress, increases the number of cupped cells and mucus production, and is associated with increased intestinal permeability and systemic inflammation. Panpan-He [51] used data from 16,257 participants from the China Health and Nutrition Survey (CHNS). These individuals were free of diabetes, but during follow-up (median duration of 9.0 years), 1097 participants developed new-onset diabetes. The authors later analyzed the dietary Zn intake and the risk of diabetes onset and found a U-shaped relationship between dietary Zn and diabetes incidence in Chinese adults, with a breakpoint of approximately 9.1 mg/day. As dietary Zn is the main source of Zn in the body, the results of our study also support the idea that Zn intake is manageable and that excessive Zn intake can cause disease. Moreover, Wolfgang Maret [52] showed that high intakes of Zn can cause Cu deficiency and that the current assumed range between safe and unsafe Zn intake is relatively narrow.

This study has several strengths. First, the samples were randomly selected from a representative clinical hospital in Northeast China with a complete quality control system in the clinical laboratory, which could ensure the comparability and stability of the detection of various nutrients. Second, the interaction between genes and nutrients and HHcy is relatively little-studied in China, which is of great significance for disease prevention. Third, we comprehensively analyzed the relationship between plasma Zn concentration and HHcy and their dose-effect and threshold-effect relationships. In the logistic regression and RCS models, we considered several covariates, including gene polymorphisms, to obtain accurate and robust results.

Nevertheless, our study has several limitations. As we considered multiple factors in the study, a larger sample size is needed to validate our findings. In addition, we could not establish a generalized causal relationship between nutrients and HHcy risk among the Chinese population, justifying a more randomized controlled trial studying plasma Zn concentration and HHcy.

## 5. Conclusions

In summary, the HHcy group had more males and a higher *MTHFR* 677TT proportion, higher BMI, and higher UA, Zn, Fe, vitamin A, and P levels. We also found a positive association between plasma Zn levels and HHcy in patients in Northeast China. The risk of HHcy was reduced with decreasing plasma Zn concentration, and the threshold value was 83.89 µmol/L. Our results highlight that individuals residing in Northeast China, especially those with the *MTHFR* 677TT polymorphism, should monitor their plasma Zn and Hcy levels.

## Figures and Tables

**Table 1:** Characteristics of the control and HHcy group participants.

Index	Control (*n* = 135)	HHcy Group (*n* = 68)	*p*-Value
Sex			0.000
Male, *n* (%)	62 (45.9%)	51 (75.0%)	
Female, *n* (%)	73 (54.1%)	17 (25.0%)	
Age, years (± SD)	24.72 ± 18.05	27.22 ± 14.84	0.057
Height, cm (± SD)	153.71 ± 19.32	164.64 ± 30.45	0.631
Weight, kg (±SD)	53.00 ± 21.89	69.20 ± 26.28	0.138
BMI ± SD	21.40 ± 5.58	24.56 ± 7.16	0.001
BMI Group			0.021
BMI < 18.5, *n* (%)	44 (32.60%)	13 (19.10%)	
18.5 ≤ BMI ≤ 23.9, *n* (%)	50 (37.00%)	28 (41.20%)	
24 ≤ BMI ≤ 28, *n* (%)	22 (16.30%)	7 (10.30%)	
BMI > 28, *n* (%)	19 (14.10%)	20 (29.40%)	
ALKP, U/L (± SD)	159.61 ± 85.03	131.89 ± 74.16	0.023
UA, mmol/L (±SD)	410.61 ± 72.17	475.43 ± 116.52	<0.001
Hcy, µmol/L (± SD)	9.26 ± 2.74	35.89 ± 20.11	<0.001
*MTHFR* 677			<0.001
CC, *n* (%)	28 (20.70%)	5 (7.40%)	
CT, *n* (%)	77 (57.00%)	28 (41.20%)	
TT, *n* (%)	30 (22.20%)	35 (51.50%)	
*MTHFR* 1269			0.083
AA, *n* (%)	94 (69.63%)	57 (83.80%)	
AC, *n* (%)	36 (26.67%)	10 (14.70%)	
CC, *n* (%)	5 (3.70%)	1 (1.50%)	
*MTRR* 66			0.853
AA, *n* (%)	70 (51.85%)	35 (51.50%)	
AG, *n* (%)	56 (41.48%)	27 (39.70%)	
GG, *n* (%)	9 (6.70%)	6 (8.80%)	

BMI, body mass index; ALKP, alkaline phosphatase; UA, uric acid; Hcy, homocysteine; HHcy, hyperhomocysteinemia; *MTHFR*, methylenetetrahydrofolate reductase; *MTRR*, methionine synthase reductase; SD, standard deviation.

**Table 2:** Comparison of Hcy concentrations among different genotypes in sex subgroups.

Genotype	*n*	Hcy Concentrations (µmol/L)			*p*-Value ^1^	*p*-Value ^2^	*p*-Value ^3^
		Total	Male	Female			
*MTHFR* 677 CC	33	11.40 ± 7.39	11.97 ± 6.33	10.86 ± 8.45	<0.001	<0.001	0.095
*MTHFR* 677 CT	105	14.22 ± 11.39	15.56 ± 10.30	12.63 ± 8.49			
*MTHFR* 677 TT	65	28.01 ± 23.54	33.38 ± 14.39	19.41 ± 9.64			
*MTHFR* 1298 AA	151	19.46 ± 18.17	23.53 ± 8.11	14.52 ± 7.97	0.196	0.107	0.911
*MTHFR* 1298 AC	46	14.54 ± 14.47	14.91 ± 8.96	14.07 ± 7.53			
*MTHFR* 1298 CC	6	13.74 ± 6.38	16.64 ± 7.97	10.84 ± 3.31			
*MTRR* 66 AA	105	17.00 ± 14.56	20.81 ± 6.79	12.49 ± 9.12	0.526	0.695	0.408
*MTRR* 66 AG	83	19.03 ± 20.36	20.99 ± 7.09	16.71 ± 9.46			
*MTRR* 66 GG	15	21.64 ± 16.42	26.19 ± 8.10	12.55 ± 7.16			

^1^ Comparison of Hcy concentrations in different subtypes of the same gene. ^2^ Comparison of Hcy concentrations in different subtypes of the same gene in male. ^3^ Comparison of Hcy concentrations in different subtypes of the same gene in female.

**Table 3:** Nutrient differences between the control and HHcy groups.

Characteristics	Control (*n* = 135)	HHcy Group (*n* = 68)	*p*-Value
Vitamin D, ng/mL (±SD)	22.55 ± 10.04	20.56 ± 8.61	0.160
Zn, µmol/L (±SD)	83.57 ± 12.79	90.54 ± 11.71	<0.001
Vitamin B12, pg/mL (±SD)	478.19 ± 241.07	266.62 ± 146.63	<0.001
fol, ng/mL (±SD)	9.76 ± 5.04	6.38 ± 3.29	<0.001
Fe, µmol/L (±SD)	15.65 ± 5.30	17.45 ± 5.03	0.020
Pb, ug/L (±SD)	24.16 ± 6.55	24.13 ± 6.42	0.970
Cu, µmol/L (±SD)	14.93 ± 3.09	14.38 ± 2.10	0.190
Ca, mmol/L (±SD)	2.34 ± 0.09	2.33 ± 0.08	0.350
Mg, µmol/L (±SD)	1.06 ± 1.50	0.93 ± 0.06	0.490
Vitamin A, pg/mL (±SD)	496.18 ± 54.47	536.08 ± 111.54	0.001
P, mmol/L (±SD)	1.39 ± 0.25	1.32 ± 0.22	0.040

Zn, zinc; fol, folic acid; Fe, iron; Pb, plumbum; Cu, copper; Ca, calcium; Mg, magnesium; P, phosphorus.

**Table 4:** Associations between Zn and the HHcy odds ratio.

Quartiles of Plasma Zn (µmol/L)					
Model	Q1	Q2	Q3	Q4	*p*-Value
	(<78.06)	(78.06–83.89)	(83.89–93.06)	(≥93.06)	
Control/HHcy	44/7	35/16	30/21	26/24	0.419
Model 1	0.172 (0.065, 0.455)	0.495 (0.220, 1.114)	0.758 (0.345, 1.665)	1 (ref.)	0.003
Model 2	0.273 (0.092, 0.813)	0.631 (0.258, 1.54)	1.131 (0.468, 2.733)	1 (ref.)	<0.001
Model 3	0.148 (0.034, 0.646)	1.159 (0.376, 3.571)	1.137 (0.386, 3.354)	1 (ref.)	<0.001

Model 1, unadjusted; Model 2, adjusted for age, sex, and body mass index; Model 3, further adjusted for vitamin B12, folic acid, and *MTHFR* C677T.

## Data Availability

Not applicable.
